# Controlling Residual Stress and Microstructure Distribution in an Invar Alloy Joint Fabricated by Oscillating Laser Welding

**DOI:** 10.3390/ma18174099

**Published:** 2025-09-01

**Authors:** Yi Jiang, Xing Liu, Suming Chen, Kun Zhou, Yanqiu Zhao, Xiaohong Zhan

**Affiliations:** 1 Avic Xi’an Aircraft Industry Group Company Ltd., Xi’an 710089, China; 2 College of Materials Science and Technology, Nanjing University of Aeronautics and Astronautics, Nanjing 211106, China

**Keywords:** invar alloy, oscillating laser welding, temperature field, residual stress, microstructure distribution

## Abstract

The efficient and high-quality welding for joining Invar alloy parts is imperative for the fabrication of composite material forming molds. The residual stress distributions and microstructural evolution during oscillating welding of Invar alloy remain inadequately characterized in the current literature, necessitating further comprehensive investigation. In this paper, laser oscillating welding with circle mode is carried out for 5 mm thick plates of Invar alloy. A finite element model for the laser oscillation welding process of Invar alloy has been established. The numerical simulations and experimental methodologies are synthetically carried out to investigate the influence of oscillating parameters on temperature field, residual stress field, and microstructure characteristics. Furthermore, the microstructural evolution of laser oscillating-welded Invar alloy is elucidated by correlating it with the characteristic distribution of the temperature field. Simulation results showed that the residual stress significantly decreases under the action of the oscillating laser. The increasing of the oscillation frequency and amplitude results in a more uniform distribution of the residual stress, and the stress peak shows a downward trend. It is indicated that the oscillation of the beam resulted in the formation of numerous fragmented fine crystals within the weld seam. Consequently, the tensile strength and elongation of the oscillating welded joint exhibit respective enhancements of 15.0% and 36.6% compared to the non-oscillating condition.

## 1. Introduction

Invar alloy, distinguished by its low thermal expansion coefficient, exhibits excellent structural stability and dimensional accuracy [1,2,3]. The aforementioned intrinsic property substantially facilitates its widespread utilization in aerospace, precision instrumentation, and the electronic industry [4,5]. The efficient and high-quality welding for the joining of Invar alloy is imperative for the successful fabrication of Invar alloy structural components. Currently, the welding methodologies employed for Invar alloy primarily encompass tungsten inert gas welding (TIG) and metal inert gas (MIG). Nevertheless, these conventional techniques are frequently associated with substantial heat input, compounded by the inherently low thermal conductivity of Invar alloy. This combination invariably leads to pronounced grain coarsening within the fusion zone, a phenomenon which consequently compromises the mechanical integrity and strength of the resultant weld joint [6]. Furthermore, the localized thermal nature inherent to the welding process induces heterogeneous thermal expansion and contraction across disparate regions of the weldment. Consequently, this disparity engenders residual internal stresses within the structure post-welding [7]. These inherent stresses, in turn, can detrimentally influence the load-bearing capacity of Invar alloy structural components when subjected to demanding service conditions.

Laser oscillating welding technology utilizes the deflection capabilities of a galvanometer scanner to facilitate intricate beam trajectories, thereby preserving the intrinsic advantages inherent to conventional laser welding. Moreover, the implementation of beam oscillation serves to extend the heat-affected zone, concomitantly mitigating the stringent assembly precision demands typically requisite for standard laser welding processes. This methodology further manifests substantial advantages in the realms of defect mitigation, microstructural optimization, and enhanced control over the ultimate performance characteristics of the weld [8,9,10,11]. Gong et al. [12] conducted narrow-gap laser oscillating welding experiments on thick aluminum alloy plates. Their findings indicated that laser oscillation stabilized the keyhole and reduced its collapse frequency, thereby enhancing welding stability. Wang et al. [13] compared weld porosity distributions resulting from three distinct beam oscillation trajectories: transverse linear, circular, and lemniscate. Their results demonstrated a significant reduction in porosity count and revealed that the oscillating laser decreased the weld depth-to-width ratio, facilitating bubble escape. Low-frequency oscillation effectively mitigates solidification cracking susceptibility, with the inhibitory effect intensifying at higher oscillation frequencies [14,15]. Yan et al. [16] investigated the influence of thermal effects on grain evolution within IN718 welded joints produced by laser oscillating welding. They observed a 40% reduction in grain size, attributable to beam oscillation. This grain refinement was attributed to the expansion of the constitutional supercooling zone and an increase in heterogeneous nucleation sites, ultimately leading to enhanced microhardness and tensile properties of the welded joint [17,18].

The complex trajectory motion of the beam spot intensifies the thermal processes and fluid dynamics within the molten pool, complicating predictions of the post-weld stress state. Recent advances in computational capabilities have established numerical simulation as an effective methodology for investigating the mechanistic effects of beam oscillation on welding processes [19,20,21]. Jiang et al. [22] developed an equivalent static heat source model for laser oscillating welding, demonstrating that beam oscillation reduces the peak temperature of the molten pool. With increasing oscillation amplitude, the transverse temperature gradient within the molten pool decreased significantly. Ke et al. [23] constructed a three-dimensional transient fluid flow model to analyze porosity formation mechanisms. Their simulations revealed that beam oscillation generates a more stable keyhole and a larger, shallower weld pool, thereby reducing keyhole collapse propensity. This effect simultaneously suppresses bubble formation and enhances bubble escape from the molten pool. Convex welds were formed with poor surface formation using linear, 8-shaped and infinity oscillating lasers. A circular oscillating laser with more uniform energy distribution favored the formation of concave weld [24,25]. Yan et al. [26] implemented a thermal–elastic–plastic finite element model for the laser oscillating welding of 316LN stainless steel, quantifying the influence of oscillation parameters on temperature and stress fields. Their results indicate that beam oscillation mitigates stress concentration at the joint, while the peak molten pool temperature decreases with higher oscillation frequencies and larger amplitudes. Laser beam oscillation effectively reduces the area of high residual tensile stress in the center of the weld [27]. Additionally, reducing welding speed at appropriate oscillation frequencies yields minimized distortion [28].

Laser oscillating welding optimizes fluid dynamics within the molten pool and modifies solidification–crystallization processes. This technique enhances process adaptability for ultra-thin structure welding, facilitates homogenization of solute distribution, and effectively suppresses porosity and solidification cracking defects [29,30]. Hagenlocher et al. [10] observed that transverse sinusoidal oscillation during welding of 6061 aluminum alloy accelerated partial solidification kinetics, promoting equiaxed grain formation and suppressing solidification cracking susceptibility. Wang et al. [31] employed a novel dual-beam oscillating laser welding technique. It was demonstrated that the welding efficiency and molten pool stability were maintained by the primary laser beam, while oscillation of the auxiliary laser induced controlled melt agitation. This synergistic interaction collectively promoted grain refinement, disrupted preferential grain growth, and facilitated the development of new crystallographic orientations. For the Invar welded joints, the microstructure of the weld seam is occupied by coarse columnar grains. The introduction of low frequency oscillating is beneficial to promoting grain refining and increasing the number of broken grains [32]. Further, beam oscillation could achieve the transformation of solidification microstructures from oriented dendrite grain to equiaxed dendrite grain [33].

The thermal history during welding and the resultant post-weld stress state constitute critical determinants of joint integrity. During laser oscillating welding, the molten pool exhibits periodic temperature distribution fluctuations. Concurrently, the joint experiences complex thermal cycles due to beam oscillation, rendering residual stress prediction particularly challenging. Furthermore, the multiplicity of process parameters in laser oscillating welding complicates isolation of individual variable effects on temperature and stress distributions through experimental methods alone. This limitation also precludes accurate characterization of transient temperature and stress field evolution throughout the welding process. In this paper, laser oscillating welding with circle mode is carried out for Invar alloy with a thickness of 5 mm. A finite element model for the laser oscillation welding process of Invar alloy has been established. The numerical simulations and experimental methodologies are synthetically carried out to investigate the influence of oscillating parameters on temperature field, residual stress field, and microstructure characteristics. Furthermore, the microstructural evolution of laser oscillating-welded Invar alloy is elucidated by correlating it with the characteristic distribution of the temperature field. This research seeks to furnish a theoretical foundation to inform the optimization of process parameters for Invar alloy laser oscillating welding within practical engineering applications.

## 2. Experimental Procedures and Analytical Methods

### 2.1. Experimental Setup and Characterization

The experimental material employed was Invar36 alloy with dimensions of 100 mm × 50 mm × 5 mm, whose chemical compositions are listed in Table 1. Before welding, the surfaces of the prepared plates were ground and then washed with acetone solution to remove the oxidation film.

**Figure 1:**
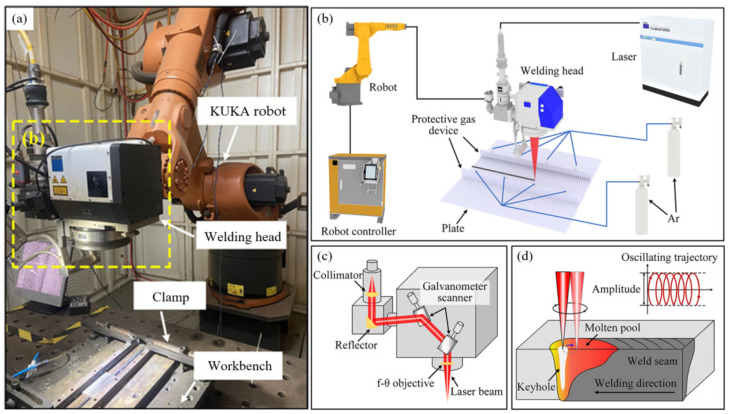
The schematic diagram of (**a**) laser oscillating welding equipment; (**b**) galvanometer scanning system; (**c**) laser oscillating welding principle; (**d**) laser oscillation welding path.

The schematic diagram of the welding setup, the structure of the galvanometer scanning system, and the principle of laser welding with beam oscillation are presented in Figure 1. The equipment used in this research was composed of a TruDis-12003 disk laser (Ditzingen, Germany), a TruDisk PFO 33 welding head (Ditzingen, Germany), a six-axis KUKA robot (Augsburg, Germany), and a corresponding protective gas device. Laser beam movement was controlled by the rotation of galvanometer scanners. The laser was collimated, reflected by galvanometer scanners, and eventually focused on the surface of the workpiece to realize the process of laser oscillating. The pipe-type protective gas device was adopted to guarantee the protection effect. The impurities were prevented from entering the molten pool by blowing 99.99% argon from the side.

The oscillating trajectory of the laser beam in this experiment was circular. The selected oscillating frequency and oscillating amplitude were varied within the range of 25–100 Hz and 0.5–2.0 mm. The values of laser power, welding speed, and defocus amount were fixed and are summarized in Table 2. In addition, a shielding gas flow of 25 L/min was used to protect the molten pool from contamination.

After welding, metallographic samples were prepared by wire cutting. The metallographic surfaces were ground with the 400–800–1000–1500–2000 grit abrasive paper, polished with the diamond polishing paste, and subsequently etched with the aqua regia (HCl:HNO_3_ = 3:1). The geometry morphology of the welded joints was observed on an optical microscope (OM, Nexcope NM900RF, Ningbo, China). Three dog-bone tensile specimens (2 mm thickness × 80 mm gauge length) were extracted perpendicular to the weld seam via wire electrical discharge machining, with the sampling plane positioned 0.5 mm below the weld crown surface. Prior to mechanical testing, all specimen surfaces were ground with 400-grit silicon carbide abrasive paper to eliminate machining artifacts, thereby mitigating potential stress concentrations from surface irregularities that could compromise testing validity. Uniaxial tensile tests were subsequently conducted at ambient temperature using a CMT5105 electromechanical universal testing system (Shenzhen, China), maintaining a constant crosshead displacement rate of 2 mm/min throughout loading. Strain measurements during tensile loading were acquired using an electronic extensometer mounted on the specimen gauge length.

### 2.2. Finite Element Simulation Modeling

**Figure 2:**
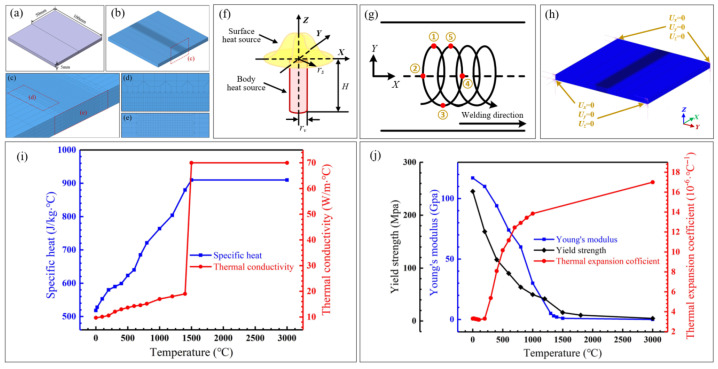
Detailed information of simulation: (**a**–**e**) geometric model and mesh model; (**f**) combined heat source model; (**g**) oscillating trajectory; (**h**) displacement constraint condition; (**i**) thermal properties of Invar alloy; (**j**) mechanical properties of Invar alloy.

The MSC Marc Mentat 2016 (64 bit) software is employed to simulate the laser oscillating welding process. The geometric model and meshing were carried out using the Hypermesh 2017 software, and the results are shown in Figure 2a–e. The dimensions of the geometric model established is consistent with the workpiece prepared in the actual welding experiment. In order to balance the computational efficiency and solution accuracy, the same mesh size cannot be used for the whole model, and the method of transitional mesh division is adopted. Since the temperature variation is mainly concentrated in the weld, the corresponding region is divided by dense meshes with a size of 0.5 mm × 0.5 mm × 0.33 mm, while the region far from the weld is not directly affected by the heat source and divided by sparser meshes. The component is ultimately meshed into 76,000 cells and 84,919 nodes.

In establishing the laser heat source model, the radiation from the plasma onto the workpiece surface is represented as a surface heat source following a Gaussian distribution. Furthermore, a cylindrical volumetric heat source is employed to simulate energy transfer in the vicinity of the keyhole, aligning with the deep penetration characteristics inherent to laser welding. Thus, a composite heat source model, integrating both surface and volumetric components, effectively simulates the heating effect of the laser beam. The schematic representation of this model is depicted in Figure 2f.

The heat flux density of the Gaussian surface heat source can be described as follows:

(1) $$q_{s}(x,y)=\frac{\alpha_{s}Q_{s}}{\pi {r_{s}}^{2}}\exp[-\frac{\alpha_{s}(x^{2}+y^{2})}{{r_{s}}^{2}}]$$

where *α_s_* is the concentration coefficient of surface heat source, *Q_s_* is the effective power of surface heat source, and *r_s_* is the radius of surface heat source.

The heat flux density of the Gaussian cylinder body heat source can be described as follows:

(2) $$q_{v}(x,y,z)=\frac{6Q_{v}(H_{v}-\beta h)}{\pi {r_{v}}^{2}{H_{v}}^{2}(2-\beta )}\exp[-\frac{3(x^{2}+y^{2})}{{r_{v}}^{2}}]$$

where *Q_v_* is the effective power of the body heat source, *H_v_* and *r_v_* are effective depth and radius of the body heat source, respectively, *β* is the attenuation coefficient of the body heat source, and *h* denotes the depth coordinate defining the position perpendicular to the material surface.

The relationship between two heat sources conforms to the equation as follows:

(3) $$Q\eta =Q_{s}+Q_{v}$$

where *Q* is the total laser power and *η* is the absorption coefficient of the heat source.

In the process of laser circular oscillating welding, the beam moves forward along the welding direction and moves in circles at a certain frequency simultaneously. The superposition of two motions determines the trajectory of laser. The change in beam location is controlled by the transformation of the local coordinate system. After substituting the trajectory equation into the heat source function, the load of the oscillated laser is realized through the compilation of subroutines. As shown in Figure 2g, the movement of the laser spot in one period is divided into four time steps for loading, and the symbol ①–⑤ represent an oscillating period.

The trajectory equation of beam circular oscillation is written as follows:

(4) $$\left\{\begin{array}{l} x\left(t\right)=x_{0}+vt+\frac{A}{2}\sin\left(2\pi ft\right)\\ y\left(t\right)=y_{0}+\frac{A}{2}\cos\left(2\pi ft\right) \end{array}\right.$$

where *x*(*t*) and *y*(*t*) are the real time coordinates of the laser spot during movement. *x*_0_ and *y*_0_ are the initial coordinates. *v* is the welding speed. *t* is the welding time. *A* and *f* are the oscillating amplitude and frequency, respectively.

The loading of the displacement constraint condition needs to restore the actual clamping situation to the maximum extent. After comprehensive consideration, the eight corners of weldment are fully fixed (as shown in Figure 2h). Three-dimensional rigid constraints are set, that is, no displacement occurs in the x, y, and z directions.

This model neglects the complex physical phenomena and chemical reactions occurring during the welding process. Specifically, it disregards the effects of metal vaporization, phase transformation latent heat, and microstructural evolution on the temperature and stress fields. Throughout the simulation, computational elements are assumed to remain in a solid state, ignoring the influence of fluid flow within the molten pool on heat transfer. Additionally, the model ignores the cooling effect of the shielding gas on the weld and disregards heat exchange between the workpiece and the workbench. The model also assumes the material is isotropic. The model takes account of material parameters that are changed as temperature changes, and the thermos-physical properties of Invar alloy are presented in Figure 2i,j. The data in the low temperature range is obtained through literature review, while that in the high temperature range is obtained through data interpolation or extrapolation.

During the welding process, the base metal melts under the heat input from the welding source. Owing to the localized heating characteristics of the source, a temperature gradient naturally arises from the weld centerline to the edges of the parent material. This gradient drives continuous heat transfer within the workpiece. This phenomenon constitutes a three-dimensional transient heat conduction problem, as described in heat transfer theory. The solution for the welding temperature field can be obtained through the analysis of the governing heat conduction equation with subsequent application of the boundary conditions. The differential heat conduction equation is derived from the principle of thermal energy conservation and Fourier’s law of heat conduction. This equation can be expressed as:

(5) $$\rho C_{p}\frac{\partial T}{\partial t}=k\left(\frac{\partial^{2}T}{\partial x^{2}}+\frac{\partial^{2}T}{\partial y^{2}}+\frac{\partial^{2}T}{\partial z^{2}}\right)+Q+\Delta H$$

where *ρ* denotes the material density, *C_p_* represents the specific heat capacity, *k* signifies the thermal conductivity, *Q* corresponds to the laser heat input, and Δ*H* denotes the phase transformation latent heat.

Achieving a complete solution for the welding temperature and stress fields necessitates not only the analysis of the heat conduction and elastoplastic constitutive equations, but also the appropriate specification of initial and boundary conditions. The initial condition is defined by setting the starting temperature of the workpiece and the ambient environment to 20 °C. In simulating the laser-oscillating welding process of Invar alloy, the boundary conditions encompass a convective–radiative heat transfer boundary condition and a heat flux boundary condition. During welding, heat exchange occurs between the workpiece surface and the surrounding air via convection and radiation. To simplify the computational model, the combined cooling effects of convection and radiation are lumped into a comprehensive heat transfer coefficient (taken as 40 W·m^−2^·K^−1^ in this study). This coefficient is applied uniformly across all external surfaces of the model. The mathematical expression for this heat transfer boundary condition is given below:

(6) $$k\frac{\partial T}{\partial x}n_{x}+k\frac{\partial T}{\partial x}n_{y}+k\frac{\partial T}{\partial x}n_{z}=\beta_{r}(T-T_{0})$$

where *n_x_*, *n_y_*, *n_z_* represents the direction cosines of the boundary normal vector, *β_r_* represents the comprehensive heat transfer coefficient, and *T*_0_ represents the temperature of the surrounding medium.

The laser heat source is implemented via a heat flux boundary condition. The welding surface heat flux and welding volumetric heat flux are applied to the corresponding element faces and elements, respectively. The heat flux density distribution is mathematically given by:

(7) $$k\frac{\partial T}{\partial x}n_{x}+k\frac{\partial T}{\partial x}n_{y}+k\frac{\partial T}{\partial x}n_{z}=q(x,y,z,t)$$

where *q*(*x*,*y*,*z*,*t*) represents the external heat input during welding.

## 3. Results and Discussion

### 3.1. Weld Morphology

**Figure 3:**
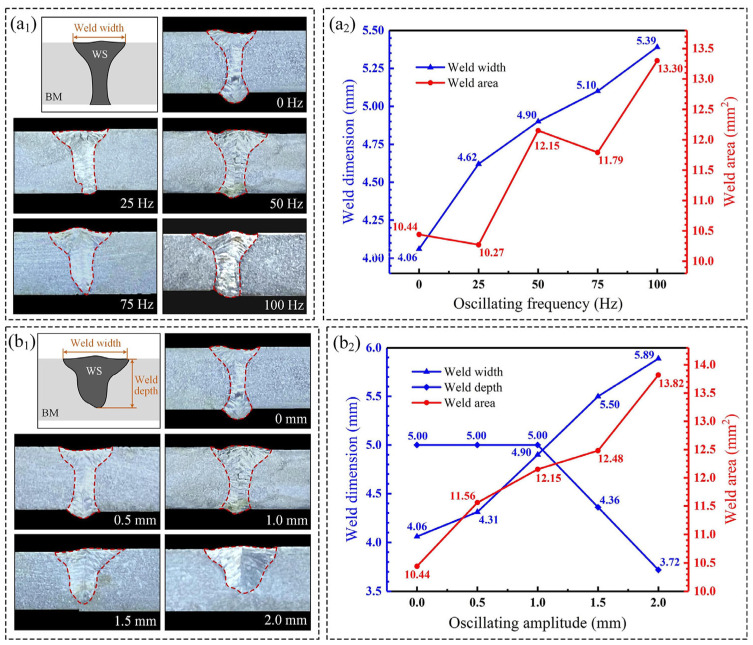
The effect of oscillating frequency and oscillating amplitude on weld morphology: (**a_1_**,**a_2_**) oscillating frequency; (**b_1_**,**b_2_**) oscillating amplitude.

Figure 3(a_1_) presents the cross-sectional weld morphology under varying oscillating frequencies (oscillating amplitude is 1.0 mm). Notably, in the absence of beam oscillation (0 Hz), weld collapse at the top surface and significant back protrusion are observed. The introduction of beam oscillation markedly increases the weld width and substantially mitigates back protrusion. At a low oscillating frequency (25 Hz), the weld top surface exhibits relative flatness. However, the low coincidence of the laser track and poor energy continuity result in the completion of molten pool solidification prior to the beam’s return. Consequently, upon its return, the beam exerts a reheating effect on the solidified weld metal, generating distinct periodic remelting features (“overlapping” traces). With further increase in oscillating frequency, the weld top surface morphology transitions, characterized by a central protrusion and depressed edges. Furthermore, weld symmetry progressively improves with increasing oscillating frequency. At the highest tested frequency (100 Hz), the degree of edge depression reaches its maximum compared to other frequencies.

As depicted in Figure 3(a_2_), the weld width is markedly greater under oscillating conditions compared to non-oscillated welding. Beam oscillation substantially increases weld width. Moreover, weld width exhibits a monotonic increase with rising oscillating frequency, whereas the depth-to-width ratio progressively decreases. No significant linear correlation is observed between weld area and oscillating frequency; instead, the area oscillates within a range as frequency changes. Specifically, at an oscillating frequency of 100 Hz, both weld width and area attain their maximum values of 5.39 mm and 13.30 mm^2^, respectively.

Figure 3(b_1_) illustrates the macroscopic weld morphology obtained under varying oscillating amplitudes (oscillating frequency is 50 Hz). The most pronounced variations are observed in weld width and penetration depth. Increasing the oscillating amplitude expands the effective laser action zone, consequently leading to a synchronous increase in weld width. Concurrently, due to energy dispersion within the enlarged interaction area, the weld penetration depth exhibits a decreasing trend. At an amplitude of 1.5 mm, the heat input becomes insufficient to achieve full penetration of the plate. Further increases in amplitude result in a continued reduction in penetration depth and a progressively flatter weld surface profile.

The values of weld width, weld depth, and weld area with oscillating amplitudes are shown in Figure 3(b_2_). With the increase in oscillating amplitude, the ratio of weld depth to width decreases, and the weld area increases. When the oscillating amplitude is 1.5 mm and 2.0 mm, the penetration depth is 87.2% and 74.4% of the plate thickness, respectively. The oscillating amplitude increases from 0 mm to 2.0 mm, the weld width increases from 4.06 mm to 5.89 mm, the weld depth decreases from 5 mm to 3.72 mm, and the weld area increases from 10.44 mm^2^ to 13.82 mm^2^.

### 3.2. Temperature Field Distribution

**Figure 4:**
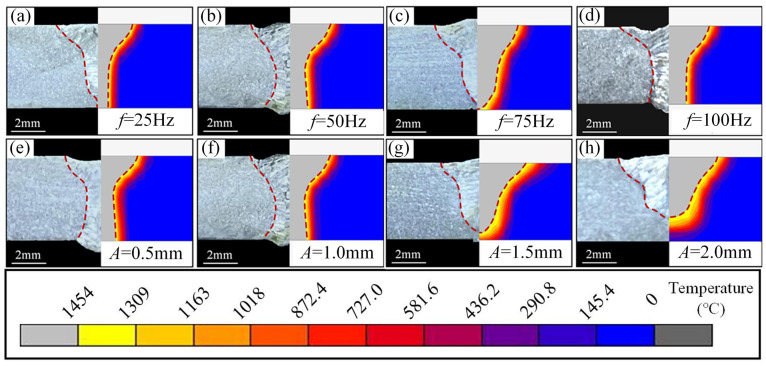
Model validation results under different welding parameters: (**a**–**d**) model validation results at oscillation frequencies of 25, 50, 75, and 100 Hz, and an oscillation amplitude of 1.0 mm; (**e**–**h**) model validation results at oscillation amplitude of 0.5, 1.0, 1.5, and 2.0 mm, and an oscillation frequency of 50 Hz.

Numerical analysis was conducted following optimization of the heat source parameters. The simulated weld pool section profile is compared with the actual weld fusion line profile to ensure the accuracy of the finite element calculation results. As shown in Figure 4, the results of the model validation found that the simulation results of the molten pool are almost consistent with the experimental results, indicating that the established model can be used for subsequent laser oscillating welding process analysis.

**Figure 5:**
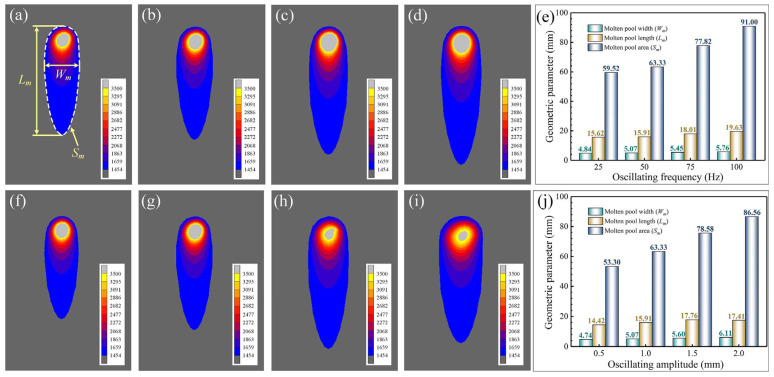
Molten pool morphology under different oscillating frequencies and oscillating amplitudes: (**a**–**d**) molten pool morphology at frequencies of 25, 50, 75, and 100 Hz, respectively, for an oscillation amplitude of 1.0 mm; (**e**) the size of the molten pool at different frequencies; (**f**–**i**) molten pool morphology at amplitudes of 0.5, 1.0, 1.5, and 2.0 mm, respectively, for an oscillation frequency of 50 Hz; (**j**) the size of the molten pool at different amplitudes.

With the oscillating amplitude fixed at 1.0 mm, the temperature field distribution was investigated at oscillating frequencies of 25 Hz, 50 Hz, 75 Hz, and 100 Hz. Figure 5a–d illustrates the characteristic morphology of the molten pool surface temperature field under these different frequencies. Dense isotherms are observed at the leading edge of the molten pool, contrasting with sparse isotherms and a discernible tailing region at the trailing edge. Increasing the frequency from 25 Hz to 50 Hz induces minimal change in the molten pool shape. However, further frequency elevation increases the absorbed laser energy, leading to significant expansion of the molten pool, particularly along its length. This observation suggests that higher oscillating frequencies intensify the molten pool tailing effect. Concomitantly, high-frequency laser oscillation enhances heat concentration, resulting in an enlarged high-temperature region at the molten pool front.

The characteristic parameters of the molten pool under different oscillating frequencies are measured, including molten pool length (*L_m_*), molten pool width (*W_m_*) and molten pool area (*S_m_*). The measurement results are shown in Figure 5e. It can be seen that the length and width of the molten pool increase synchronously with the increase in the oscillating frequency. When the oscillating frequency increases from 25 Hz to 50 Hz, the length and width of the molten pool only increased by 0.29 mm and 0.23 mm. Continuing to increase the oscillating frequency from 50 Hz to 75 Hz and from 75 Hz to 100 Hz, the length of the molten pool increased by 2.1 mm and 1.62 mm, and the width of the molten pool increased by 0.38 mm and 0.31 mm. The change trend of the molten pool area with the oscillating frequency is similar. At the oscillating frequency of 100 Hz, the molten pool area reaches 91.00 mm^2^.

The fixed oscillating frequency is 50 Hz, and the distribution of temperature field is studied when the oscillating amplitude is 0.5 mm, 1.0 mm, 1.5 mm, and 2.0 mm, respectively. Figure 5f–i show the simulation results of the surface morphology of the molten pool under different oscillating amplitudes. The results show that the width of the front molten pool increases significantly with the increase in the amplitude. At the same time, the isotherms here are sparser, and the area of the high temperature zone in the front end decreases gradually. When the oscillating amplitude is from 0.5 mm to 1.5 mm, the overall size of the molten pool is expanded, and obvious tailing phenomena can be observed at the end of the molten pool. When the oscillating amplitude is 2.0 mm, the tailing effect of the weld pool temperature field is alleviated. It is expected that the length of the molten pool will continue to decrease with the increase in the oscillating amplitude. The quantitative measurement of geometric parameters of the molten pool under different oscillating amplitudes is carried out and the results are shown in Figure 5j. It can be seen that with the oscillating amplitudes increasing from 0.5 mm to 2.0 mm, the width of the molten pool increases from 4.47 mm to 6.11 mm, and the area increases from 53.30 mm^2^ to 86.56 mm^2^. The length of the molten pool increases first and then decreases amplitude. The maximum value is at 1.5 mm oscillating amplitude.

**Figure 6:**
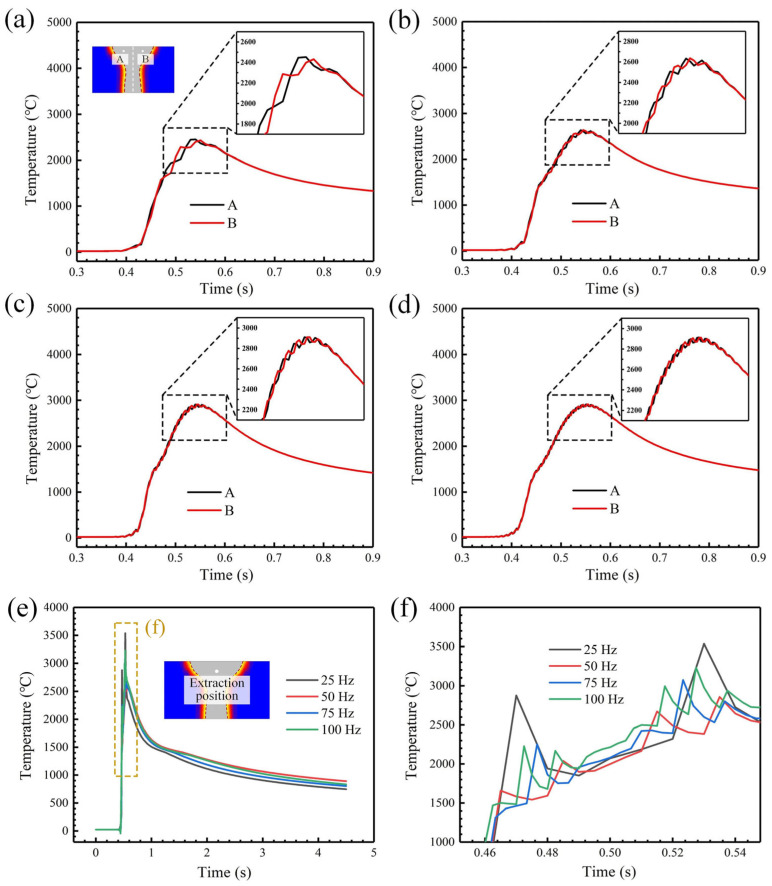
Thermal cycle curve at different positions of the model under different oscillating frequencies, and an oscillation amplitude of 1.0 mm: (**a**) 25 Hz; (**b**) 50 Hz; (**c**) 75 Hz; (**d**) 100 Hz; (**e**) thermal cycle curve at the molten pool center; (**f**) enlarged image of fluctuation area.

Figure 6 is the temperature history curve at different positions in the molten pool under different oscillating frequencies for a fixed value of oscillation amplitude of 1.0 mm. With the increase in oscillating frequency, the number of wave peaks on the curve increases significantly, and the temperature fluctuation phenomenon is more severe. Comparing the welding thermal cycles of node A and B, it can be found that the difference is mainly reflected in the heating stage, and the curves basically coincide in the cooling stage. In addition, the greater the oscillating frequency, the more similar the thermal process experienced by two nodes. The curve shape is gradually approaching, indicating that the temperature field is more uniform.

We extract the thermal cycle curve of the molten pool center, and the results are shown in Figure 6e,f. When the oscillating frequency is 25 Hz, the temperature fluctuation range is the widest. Because the average energy density of the heat source is the highest at this time, the node experiences the highest peak temperature of 3536 °C, and the peak temperature reaches the lowest value at 50 Hz. After further increasing the oscillating frequency, the peak temperature rises again. This may be due to the high energy overlap when the beam moves at high frequency, resulting in a certain amount of heat accumulation, while the thermal conductivity of Invar alloy is poor, causing an increase in the peak temperature.

**Figure 7:**
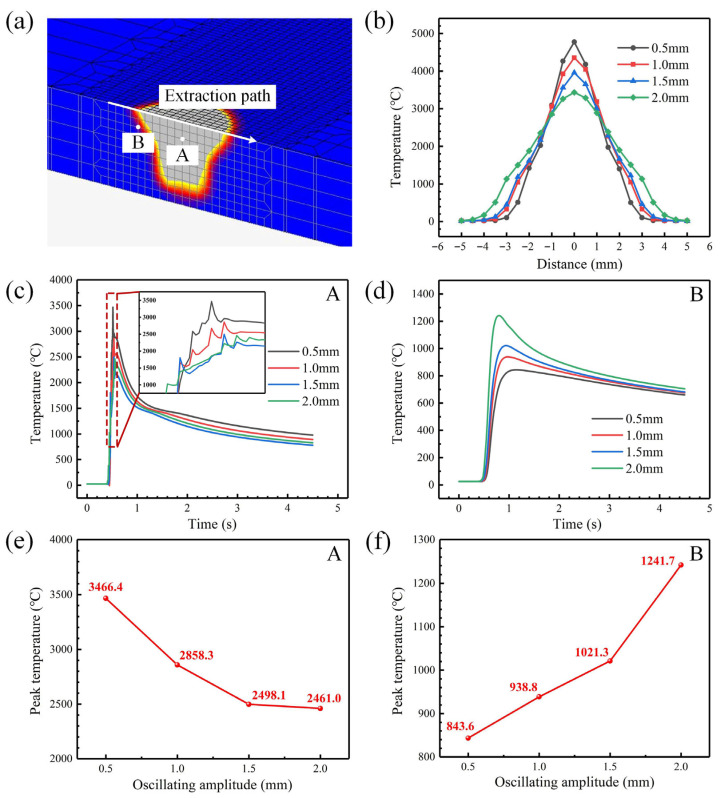
Temperature distribution and thermal cycle characteristics under different oscillating amplitudes and an oscillation frequency of 50 Hz: (**a**) temperature distribution and node position; (**b**) temperature distribution curve (**c**) thermal response curve of node A; (**d**) thermal response curve of node B; (**e**) peak temperature variation in node A; (**f**) peak temperature variation in node B.

The temperature change curve under different oscillating amplitudes is analyzed for a fixed value of oscillation frequency of 50 Hz. According to Figure 7b, it can be seen that the temperature distribution of the molten pool surface is asymmetric. With the increase in oscillating amplitude, the peak temperature and transverse temperature gradient continue to decrease. When the oscillating amplitude increased from 0.5 mm to 2.0 mm, the peak temperature decreased by 28.1% and the maximum transverse temperature gradient decreased by 55.4%. In order to study the influence of oscillating amplitude on the temperature history at different positions of the joint, the thermal cycle curves of node A and B shown in Figure 7a are extracted. As presented in Figure 7c, node A undergoes frequent temperature fluctuations. The superposition of multiple laser thermal effects causes the peak temperature to rise gradually. The larger the oscillating amplitude, the longer the duration of temperature fluctuations. The thermal cycle curve of node B is different from that of node A. There is only one peak. According to the peak temperature under different oscillating amplitudes in Figure 7e,f, with the increase in oscillating amplitude, the laser energy is more dispersed, and the peak temperature of node A gradually decreases. When the oscillating amplitude is from 0.5 mm to 2.0 mm, the peak temperature at node A decreases by 29%, while the peak temperature at node B rises from 843.6 °C to 1241.7 °C, which is due to the increase in the range of the heat source; the laser is closer to the selected node and causes a higher peak temperature under the effect of heat conduction.

### 3.3. Residual Stress Field Distribution

**Figure 8:**
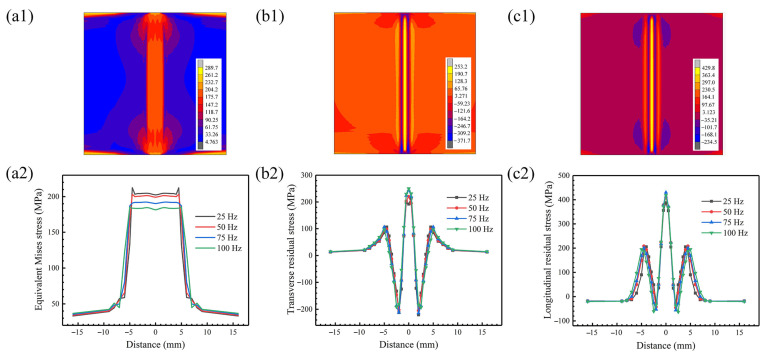
Stress field contours and stress profile at the weldment surface: (**a1**,**a2**) equivalent residual stress; (**b1**,**b2**) transverse residual stress; (**c1**,**c2**) longitudinal residual stress. Note that the scales are different.

Figure 8 shows the stress contours at the weldment surface and the stress profile under different oscillating frequencies for an oscillation amplitude of 1.0 mm. The results show that the equivalent residual stress is mainly concentrated in the weld zone. There is an obvious stress plateau, and the stress drops sharply away from that region. The oscillating frequency is from 25 Hz to 100 Hz, the stress concentration area is gradually expanded, and the corresponding stress value is gradually reduced. This is because the energy density distribution is more uniform after the oscillating frequency increases, and the uniformity of the temperature field is improved, resulting in the reduction in residual stress.

The oscillating frequency does not affect the form of stress distribution, and the trend of the transverse and longitudinal residual stresses is similar. After being away from the weld center, it undergoes the state transition of tensile stress-compressive stress-tensile stress, and is subjected to transverse tensile stress and longitudinal compressive stress at the edge of the weldment, respectively. There are two peaks of the transverse stress distribution curve. The first peak is located at the weld center. The distance between the second peak and the weld center increases, but the corresponding value of stress decreases with the increase in the oscillating frequency. In addition, the peak compressive stress is negatively correlated with the oscillating frequency. The oscillating frequency increases from 25 Hz to 100 Hz, the peak tensile stress increases from 214.3 MPa to 250.0 MPa, and the peak compressive stress decreases from −212.3 MPa to −193.3 MPa.

**Figure 9:**
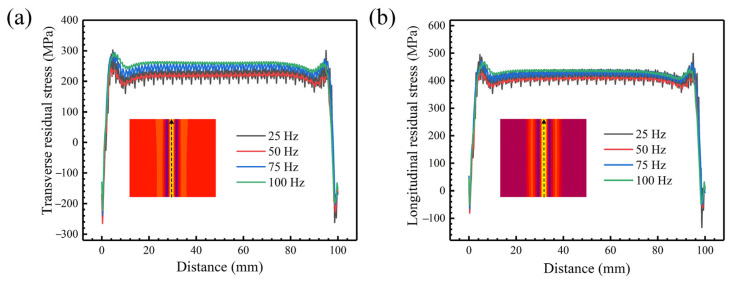
Transverse and longitudinal residual stress along the weld axis under different oscillating frequencies and an oscillation amplitude of 1.0 mm: (**a**) transverse residual stress; (**b**) longitudinal residual stress.

Figure 9 shows the stress profile along the weld direction under different oscillating frequencies and an oscillation amplitude of 1.0 mm. It can be seen that the fluctuation of transverse residual stress is significantly higher than the longitudinal residual stress. With the increase in the oscillating frequency, the number of peaks increases, indicating that the frequency of stress fluctuation increases. The range of stress fluctuation generally decreases, and the stress is basically stable at 100 Hz. In addition, the average transverse and longitudinal stresses are the lowest at the oscillating frequency of 50 Hz and the highest at the oscillating frequency of 100 Hz.

**Figure 10:**
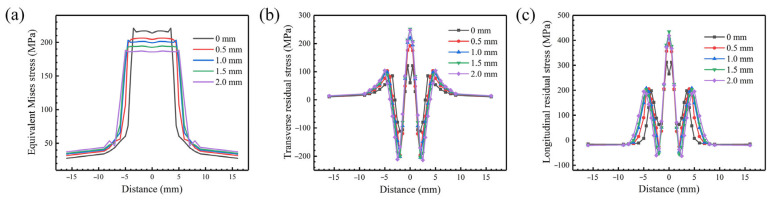
Stress profile at the weldment surface: (**a**) equivalent residual stress; (**b**) transverse residual stress; (**c**) longitudinal residual stress.

The effect of oscillating amplitude on the equivalent residual stress of the weldment surface is presented in Figure 10a for an oscillation frequency of 50 Hz. After beam oscillation, the stress plateau becomes smoother, the peak stress decreases gradually, and the stress gradient decreases significantly with the increase in oscillating amplitude. When the oscillating amplitude is 2.0 mm, the peak stress decreases by 20.8% and the plateau range expands by 41.4% compared with that without oscillation. After the oscillating amplitude is increased, the stress concentration range of weldment increases synchronously, and the maximum value of stress continues to decrease. The oscillating amplitude increases from 0.5 mm to 2.0 mm, and the peak stress decreases from 206.18 MPa to 187.09 MPa. Figure 10b,c show the transverse and longitudinal residual stress distributions on the surface of weldment, respectively. It can be seen that the stress distribution characteristics are different when the beam does not oscillate. The peak tensile stress is not located in the weld center. At the same time, the longitudinal tensile stress after being far from the center will drop sharply but not be converted into compressive stress, which indicates that under the condition of beam oscillation, the extrusion effect caused by the surrounding area is stronger after heating and expansion. In the process of superposition and cancelation of tensile stress and compressive stress, compressive stress is dominated. In addition, the peak horizontal and longitudinal stresses are relatively high when the heat source oscillates, and its value will increase with the increase in oscillating amplitude.

**Figure 11:**
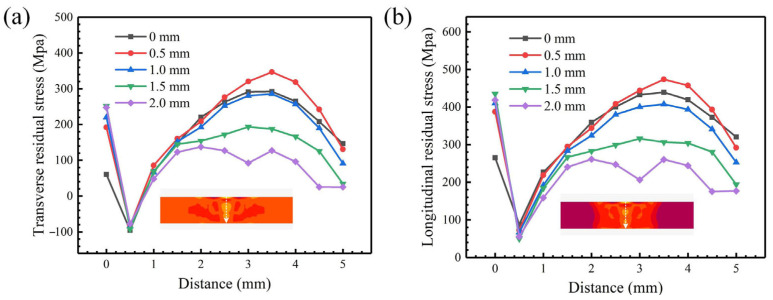
Transverse and longitudinal residual stress along the thickness direction of the weldment under different oscillating amplitudes and an oscillation frequency of 50 Hz: (**a**) transverse residual stress; (**b**) longitudinal residual stress.

Figure 11 shows the distribution curve of residual stress inside the weldment. It can be found that the stress level decreases with the increase in oscillating amplitude. When the oscillating amplitude is 0.5 mm, the average stress value is higher than that without oscillation. In addition, the residual stress under other oscillating amplitudes is relatively low. When the oscillating amplitude is 2.0 mm, the peak transverse and longitudinal stress are the lowest, reaching 137.5 MPa and 261.3 MPa, respectively.

**Figure 12:**
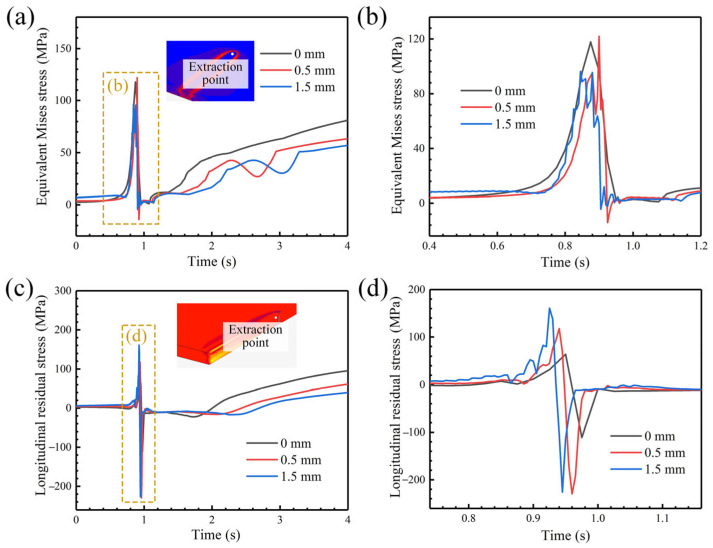
The effect of oscillating amplitude on stress history curve: (**a**) change in equivalent stress with time; (**b**) enlarged image of fluctuation area in (**a**); (**c**) change in longitudinal stress with time; (**d**) enlarged image of fluctuation area in (**c**).

The trend of the equivalent stress with time under different oscillating amplitudes is analyzed for an oscillation frequency of 50 Hz, and the stress history curve is shown in Figure 12. Before the heat source arrives, the stress is expressed as tensile stress, and when the heat source moves to the point, the metal is heated to expand and the surrounding materials are squeezed, so the stress state changes to compressive stress. The liquid metal is rapidly cooled and solidified after the heat source passes, and it is constrained by the surroundings to form tensile stress, and then the stress increases until stable. In particular, due to the repeated heating and cooling process under the action of beam oscillation, the constraint effect caused by the surrounding area during expansion and contraction changes constantly, resulting in the occurrence of stress fluctuation. In the subsequent stage of tensile stress growth, the stress growth rate is relatively slow. It can be found from Figure 12b that the stress fluctuation range is the largest when the oscillating amplitude is 0.5 mm. As shown in Figure 12c,d, when the heat source does not oscillate, its instantaneous longitudinal tensile stress and compressive stress values are the smallest, but in the subsequent cooling process, the stress growth rate is the fastest, and the stress level is the highest when it tends to be stable.

### 3.4. Microstructure and Tensile Property

**Figure 13:**
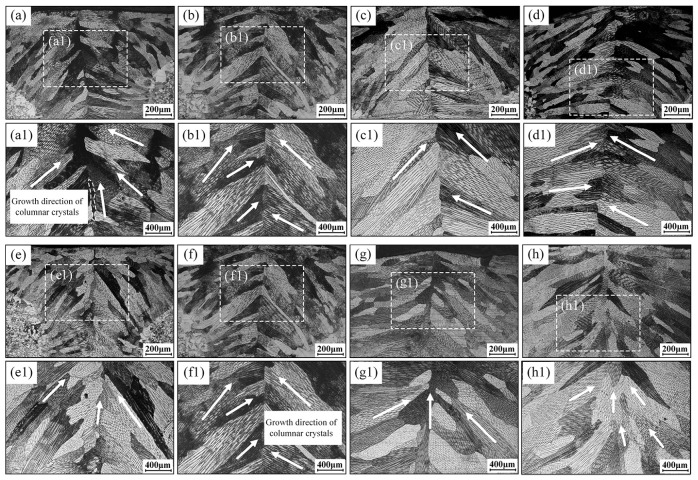
Microstructural morphology at weld centerline under varied beam oscillation frequencies and amplitudes: (**a**,**a1**) oscillation frequency of 25 Hz and an oscillation amplitude of 1.0 mm; (**b**,**b1**) oscillation frequency of 50 Hz and an oscillation amplitude of 1.0 mm; (**c**,**c1**) oscillation frequency of 75 Hz and an oscillation amplitude of 1.0 mm; (**d**,**d1**) oscillation frequency of 100 Hz and an oscillation amplitude of 1.0 mm; (**e**,**e1**) oscillation amplitude of 0.5 mm and an oscillation frequency of 50 Hz; (**f**,**f1**) oscillation amplitude of 1.0 mm and an oscillation frequency of 50 Hz; (**g**,**g1**) oscillation amplitude of 1.5 mm and an oscillation frequency of 50 Hz; (**h**,**h1**) oscillation amplitude of 2.0 mm and an oscillation frequency of 50 Hz.

Figure 13 presents the microstructures at the weld centerline under varying oscillation frequencies and amplitudes. The results demonstrate that due to limited oscillation amplitudes, steep thermal gradients persist across all frequencies, maintaining predominant columnar grain growth. However, compared to continuous columnar grains without oscillation, the oscillated specimens exhibit fragmented columnar structures with occasional equidimensional grains. Overall columnar grain length decreases, revealing internal cellular dendrites of varying sizes and orientations, predominantly exhibiting short-range morphologies with reduced dimensions. At the oscillation frequency of 25 Hz, significant thermal field heterogeneity and abrupt gradient transitions result in disordered columnar growth, including upward-oriented grains. Increasing oscillation frequency moderately enhances crystallographic alignment. When oscillation amplitude reaches 0.5 mm, it promotes heterogeneous nucleation. This yields fine equiaxed grains at the weld center containing shortened dendritic arms, with most growing toward the weld crown. At an amplitude of 1.0 mm, the increased pool width enlarges columnar grains while reducing fine central grains, yielding abundant long-range dendrites. Further increasing the amplitude to 1.5 mm reduces thermal gradients, facilitating quasi-equiaxed grain formation at the weld center with dominant short-range dendritic substructures. Grain growth directions become more randomized in the upper region. At an amplitude of 2.0 mm, unlike full-penetration conditions, bottom columnar grains develop horizontally or downward. Concurrently, upward-growing grains emerge internally (Figure 13(h1)), exhibiting fine dimensions with short-range dendrites transitioning toward equiaxed dendritic morphologies.

**Figure 14:**
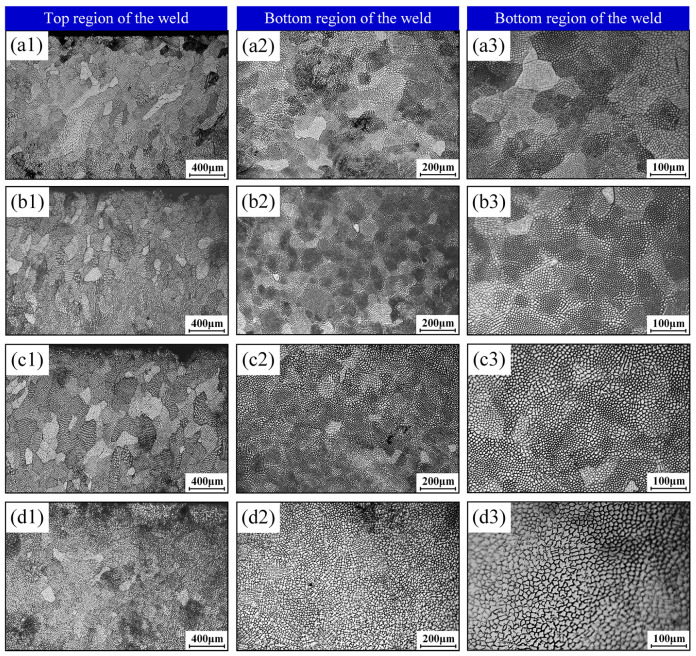
Microstructural morphology at weld top and bottom regions under varied beam oscillation frequencies and an oscillation amplitude of 1.0 mm: (**a1**–**a3**) 25 Hz; (**b1**–**b3**) 50 Hz; (**c1**–**c3**) 75 Hz; (**d1**–**d3**) 100 Hz.

Figure 14 displays grain morphology at the weld crown and root under varying oscillation frequencies and an oscillation amplitude of 1.0 mm. Results reveal equiaxed grains dominating near the top surface of the crown region, transitioning downward to columnar morphology, while the root region reverts to equiaxed dendritic structures. Longitudinal sections confirm that these equiaxed dendritic characteristics universally define the substructures, corresponding to cross-sections of columnar dendrites and cellular dendritic arms. Increasing oscillation frequency reduces columnar grain dimensions and enhances microstructural homogeneity. Notably, substructures at low-to-medium frequencies comprise equiaxed dendrites with stunted secondary arms and diminished primary dendrite arm spacing. At 100 Hz, equiaxed dendrites near the weld surface exhibit pronounced secondary branching, indicating slower solidification rates that prolong high-temperature residence, enabling secondary arms to grow until mutual impingement occurs. Substructure density per unit area initially increases then decreases with rising frequency, implying substructure size follows an inverse trend—minimizing at 50 Hz where density peaks at approximately fivefold that of 100 Hz. Identical coarsening patterns emerge across all three weld cross-sections above 75 Hz, with significant grain coarsening observed at 100 Hz.

**Figure 15:**
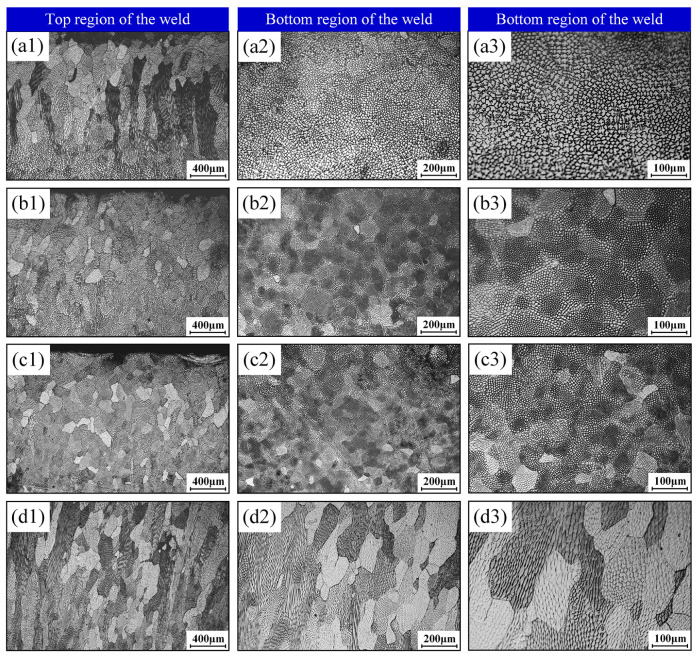
Microstructural morphology at weld top and bottom regions under varied beam oscillation amplitudes and an oscillation frequency of 50 Hz: (**a1**–**a3**) 0.5 mm; (**b1**–**b3**) 1.0 mm; (**c1**–**c3**) 1.5 mm; (**d1**–**d3**) 2.0 mm.

Figure 15 illustrates microstructural characteristics in longitudinal weld sections under varying oscillation amplitudes and an oscillation frequency of 50 Hz. At 0.5 mm and 2.0 mm amplitudes, distinct columnar grain zones emerge in crown regions due to grains exhibiting steep upward growth orientations. Specifically, the 0.5 mm condition features elongated columnar grains accompanied by coarsened substructures in the root zone, attributable to concentrated heat input energy. For an amplitude of 2.0 mm, partially massive columnar grains form with interspersed fine crystallites, revealing randomized crystallographic orientations in longitudinal sections and pronounced microstructural heterogeneity along the welding direction. Conversely, at intermediate amplitudes of 1.0 mm and 1.5 mm, homogeneous microstructures prevail without long-range columnar growth. This uniformity indicates minimized inclination angles of columnar grain growth relative to the weld axis.

**Figure 16:**
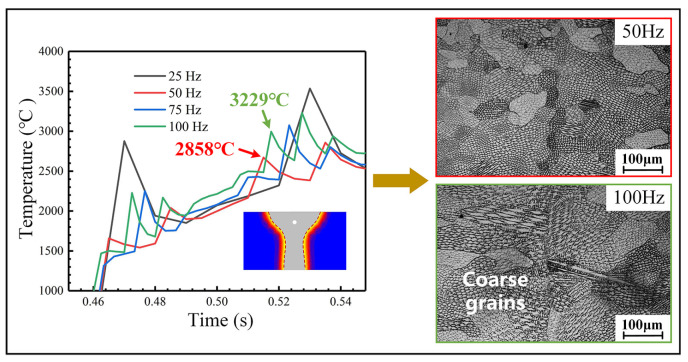
Thermal field simulation and corresponding metallographic analysis under varied oscillation frequencies for an oscillation amplitude of 1.0 mm.

Beam oscillation accelerates heat exchange in the molten pool, preventing weld metal overheating and thereby inhibiting excessive grain growth, which moderately reduces grain size. However, high-frequency oscillation causes substantial overlapping of heat source energy, leading to heat accumulation within the molten pool. As shown in Figure 16, when oscillation frequency increases from 50 Hz to 100 Hz, grain coarsening occurs due to the poor heat dissipation capacity of Invar alloy, consistent with prior thermal simulation results. The peak temperature at the molten pool center initially decreases then increases with rising oscillation frequency. Nevertheless, compared to non-oscillated conditions, fragmented fine grains are observed throughout the weld cross-section under all oscillation frequencies. Columnar grain growth becomes discontinuous due to molten pool agitation, exhibiting significantly reduced lengths.

**Figure 17:**
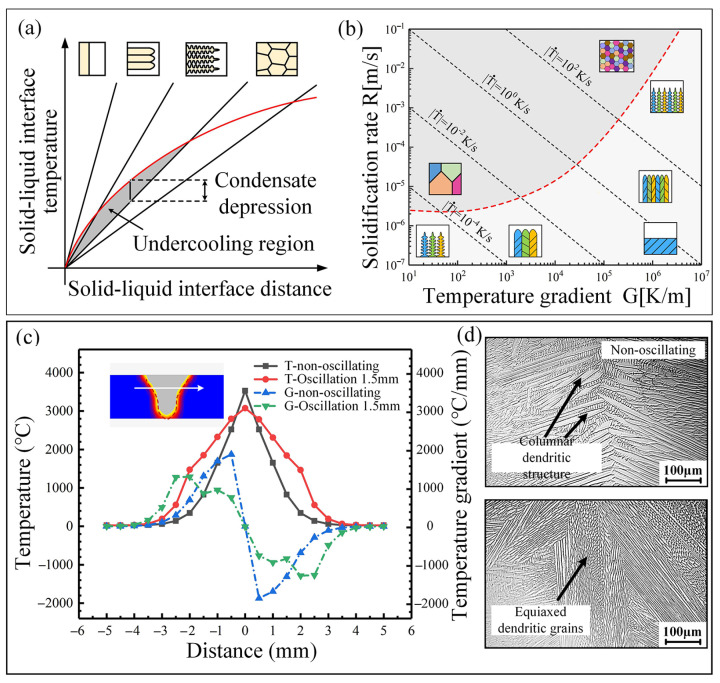
Solidification condition effects on microstructural characteristics: (**a**) schematic: constitutional supercooling and crystalline morphology; (**b**) thermal gradient (G) and solidification rate (R) governing structural evolution; (**c**) temperature and thermal gradient traverse profiles; (**d**) weld substructure morphology.

Increasing oscillation amplitude to 1.5 mm promotes equiaxed grain formation at the weld center. Beyond dendrite fragmentation mechanisms, this phenomenon aligns with solidification theory principles. The crystalline morphology depends on the thermal gradient (G) and solidification rate (R), where constitutional supercooling determines transition modes. As shown in Figure 17a, increasing undercooling drives substructure evolution: planar → cellular → cellular dendritic → equiaxed morphologies. Figure 17b demonstrates the influence of the G/R ratio on microstructure development. Lower G/R values shift solidification from columnar to equiaxed growth. High-amplitude oscillation significantly alters thermal distributions, where modified temperature gradients profoundly impact substructural morphology. Figure 17c compares simulated surface temperature distributions and thermal gradients under static and oscillated conditions (1.5 mm amplitude and an oscillation frequency of 50 Hz). Beam oscillation substantially reduces thermal gradients, expanding constitutionally supercooled zones. This enhanced undercooling facilitates homogeneous nucleation within the liquid phase. The resulting nuclei grow freely, forming equiaxed dendrites with isotropic dimensions (Figure 17d). These equiaxed grains impede columnar growth while promoting grain refinement. Higher oscillation amplitudes intensify thermal gradient fluctuations, causing dendrite truncation when growth deviates from the maximum thermal gradient direction. Subsequent amplitude increases further reduce thermal gradients, increasing equiaxed grain formation at the weld centerline.

**Figure 18:**
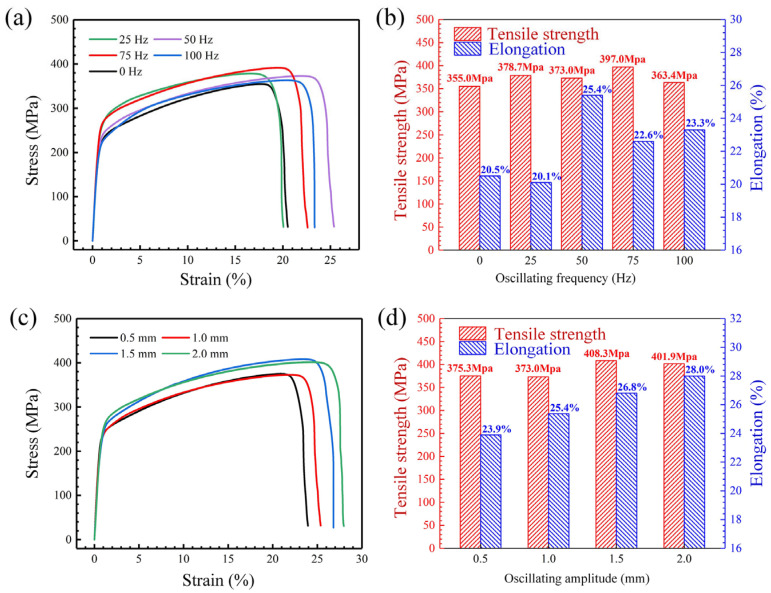
Tensile properties of welded joints under laser oscillation: frequency and amplitude: (**a**) stress–strain curves under varied oscillation frequencies and an oscillation amplitude of 1.0 mm; (**b**) tensile strength and elongation under oscillation frequency; (**c**) stress–strain curves under varied oscillation amplitudes and an oscillation frequency of 50 Hz; (**d**) tensile strength and elongation under oscillation amplitude.

Figure 18a,b present tensile test results of welded joints under varied oscillation frequencies and an oscillation amplitude of 1.0 mm. The stress–strain curves in Figure 18a exhibit no distinct yield platforms, with all curves overlapping completely during the elastic deformation stage. As quantified in Figure 18b, laser-welded joints demonstrate tensile strength and elongation of 355.0 MPa and 20.5%, respectively. Beam oscillation universally enhances both properties: tensile strength fluctuates between 363 and 397 MPa across 25–100 Hz frequencies, achieving a maximum 11.8% improvement versus non-oscillated specimens. Notably, grain coarsening at 100 Hz results in the lowest strength, while minimal elongation (20.1%) occurs at 25 Hz. Figure 18c,d characterize amplitude effects on mechanical properties for an oscillation frequency of 50 Hz. Increasing amplitude elevates tensile strength despite minor fluctuations and progressively improves elongation from 23.9% (0.5 mm) to 28.0% (2.0 mm). At 1.5 mm and 2.0 mm amplitudes, equiaxed dendritic structures at weld centers drive tensile strength to 408.3 MPa (+15.0%) and 401.9 MPa (+13.2%), respectively. Maximum elongation enhancement reaches 36.6% compared to static welding.

## 4. Conclusions

The thermal–mechanical coupling model of the laser oscillating welding process of Invar alloy is constructed, and the temperature field and stress field are solved and analyzed. The temperature field evolution and the residual stress distribution of weldment is revealed. The influence of oscillating parameters on the microstructure and mechanical properties of welded joints were deeply investigated. The conclusions are drawn as follows:

(1) The increasing of the oscillating frequency enhances the homogeneity of the temperature field. Concomitantly, the thermal histories at symmetrically positioned nodes converge, while the peak temperature at the molten pool center exhibits an initial decrease followed by an increase. Elevating the oscillating amplitude induces significant expansion of the molten pool. Furthermore, as the amplitude increases from 0.5 mm to 2.0 mm, the temperature gradient across the molten pool surface undergoes a continuous reduction, accompanied by a 29.0% decrease in the peak temperature at the monitored internal node.
(2) An augmentation in either the oscillating frequency or amplitude demonstrably enhances the homogeneity of the equivalent stress distribution across the weld surface, concomitantly mitigating the maximum stress intensity observed. Specifically, along the centerline of the weld surface, pronounced fluctuations are observed in both transverse and longitudinal residual stresses. The range of these stress fluctuations diminishes with increasing oscillating frequency. Furthermore, the average stress level along this path is relatively low at an oscillating frequency of 50 Hz. Increasing the oscillating amplitude also contributes to a reduction in internal stress within the weldment.
(3) The higher oscillation frequencies promote homogenized laser energy distribution and welding thermal fields, thereby improving microstructural uniformity throughout the weld. With increasing oscillation amplitude, a significant reduction in molten pool temperature gradient expands the constitutional supercooling zone, providing favorable conditions for equiaxed dendrite nucleation and growth. Consequently, isotropic near-equiaxed grains develop at the weld center, exhibiting homogeneous equiaxed dendritic structures internally. The tensile strength and elongation of the oscillating welded joint exhibit respective enhancements of 15.0% and 36.6% compared to the non-oscillating condition.

Subsequent investigations stemming from this work should prioritize three interconnected dimensions: The primary focus entails conducting high-fidelity, multiphysics numerical model validation through integration with in situ temperature and stress field measurements acquired during the welding thermal cycle. Further efforts should leverage advanced characterization techniques—such as electron backscatter diffraction (EBSD) and in situ synchrotron radiation—to fundamentally elucidate the grain evolution kinetics and phase transformation mechanisms under thermo-mechanical coupling. Concurrently, research should expand to novel laser processing modalities, including asymmetric oscillation waveforms and multi-frequency modulation, while exploring their process compatibility with specialized material systems like nickel-based superalloys and emerging high-entropy alloys. This integrated approach aims to establish predictive process–microstructure–property relationships for precision manufacturing.

## Figures and Tables

**Table 1:** Chemical compositions of Invar alloy (wt.%).

**Material**	C	P	S	Si	Mn	Ni	Fe
**Invar36**	≤0.05	≤0.02	≤0.02	≤0.3	≤0.6	35.0–37.0	Bal.

**Table 2:** Laser oscillating welding parameters in this experiment.

Parameter	Value
Laser power (W)	4500
Welding speed (m/min)	1.2
Defocus amount (mm)	0
Oscillating frequency (Hz)	25, 50, 75, 100
Oscillating amplitude (mm)	0.5, 1.0, 1.5, 2.0

## Data Availability

The original contributions presented in this study are included in the article. Further inquiries can be directed to the corresponding author.
